# Integrated Gut Microbiota and Metabolome Analysis Reveals Dysbiosis and Metabolic Disturbances in Diarrhea of the Complex-Toothed Flying Squirrel (*Trogopterus xanthipes*): Possible Mycotoxin-Induction?

**DOI:** 10.3390/toxins18080340

**Published:** 2026-08-03

**Authors:** Lifeng Che, Bichen Miao, Lijuan Suo, Jie Tang

**Affiliations:** Shaanxi Key Laboratory of Qinling Ecological Security, Shaanxi Institute of Zoology, Xi’an 7010032, China; chelifeng@ms.xab.ac.cn (L.C.);

**Keywords:** complex-toothed flying squirrel (*Trogopterus xanthipes*), diarrhea, gut microbiota, fecal metabolome, microbial–metabolic interaction

## Abstract

The gut microbiota and its metabolic functions are critical for intestinal homeostasis, but their integrated responses to diarrhea remain poorly understood in non-model mammals, such as the complex-toothed flying squirrel (*Trogopterus xanthipes*). Here, we collected fecal samples from six captive animals with diarrhea and six healthy controls; the diarrheal animals had been provided with moldy corn, but mycotoxin levels were not measured. Fecal microbiota was profiled by full-length 16S rRNA sequencing and untargeted metabolomics with integrative analyses. Diarrheal animals showed significantly decreased fecal microbial alpha-diversity and distinct community separation. LEfSe analysis identified *Lawsonia intracellularis* and *Streptococcus macedonicus* as the most enriched taxa in the diarrhea group. Untargeted metabolomics revealed 2216 differential metabolites (1931 downregulated), primarily enriched in steroid hormone biosynthesis, arachidonic acid metabolism, and bile secretion pathways. Procrustes and Mantel tests confirmed significant microbiome–metabolome concordance (M^2^ = 0.422, *p* < 0.001). Spearman correlation networks showed that *L. intracellularis* was positively correlated with multiple differential metabolites, while *S. macedonicus* exhibited predominantly negative correlations. These findings suggest that diarrhea in *T. xanthipes* may be associated with moldy corn exposure and is accompanied by gut dysbiosis and metabolic disturbances. *L. intracellularis* and *S. macedonicus* may be associated with disease progression through pathways linked to lipid and bile acid metabolism and inflammation. This study provides novel microbial–metabolic insights into diarrhea in a medicinal mammal, supporting disease prevention and healthy breeding.

## 1. Introduction

The complex-toothed flying squirrel (*Trogopterus xanthipes*), a rare rodent species endemic to China, represents the exclusive origin animal of the traditional Chinese medicinal material “Wulingzhi” (Faeces Trogopteri) [1,2], which has been widely used for promoting blood circulation, relieving pain, and resolving blood stasis [3,4]. Beyond its medicinal value, this species holds considerable ecological and conservation significance; however, captive breeding programs have been increasingly challenged by various health issues. While modern pharmacological studies have demonstrated that gut microbiota is critical for the host’s adaptation to its unique diet of *Platycladus orientalis* [5,6], the pathophysiological mechanisms underlying diarrhea in this species remain poorly understood.

Diarrhea, a common clinical syndrome in captive animals, is often triggered by dietary stressors such as moldy feed—a frequent issue in artificial rearing. Homeostasis of the gut microbiota and the functional stability of its metabolic network are fundamental to intestinal health [7]. Gut microbiota participates extensively in host material metabolism, energy supply, and immune regulation [8,9]. Under exogenous mycotoxin challenges, dysbiosis characterized by reduced microbial diversity and changed community composition is a core driving factor in the onset and progression of diarrhea, intestinal inflammation, and systemic tissue damage [10,11,12]. Furthermore, microbial disruption further destabilizes host metabolic networks, perturbs core metabolic pathways, and ultimately exacerbates pathological damage [13,14]. Despite these well-established links in model organisms and livestock, the dynamic responses of the gut microbiome to diarrheal perturbations in non-model wildlife species such as *T. xanthipes* remain largely uncharacterized, except for a few studies that have preliminarily characterized the composition of the normal gut microbiota in healthy *T. xanthipes* [6,15]. Beyond alterations in microbial community structure, the remodeling of host metabolic homeostasis represents another critical pathological manifestation of diarrhea. Gut microbiota dysregulation modulates host metabolic profiles via the gut-liver axis [16]. This disruption often manifests as significant alterations in key metabolic pathways, including lipid metabolism, amino acid metabolism, arachidonic acid metabolism, and bile acid secretion [17,18,19]. The dysregulation of this “microbiota-metabolism” axis has been confirmed as a central pathological mechanism in various digestive diseases. Untargeted metabolomics, offering unbiased and high-throughput profiling, has emerged as a powerful tool for deciphering such complex disturbances [20]. Nevertheless, in non-model wildlife species such as *T. xanthipes*, the microbial drivers, metabolic signatures, and their functional interplay remain virtually unexplored. While 16S rRNA sequencing can delineate taxonomic shifts, it fails to capture functional metabolic outcomes; conversely, metabolomics alone cannot identify the microbial initiators of those changes. We therefore hypothesized that moldy corn exposure disrupts gut microbial homeostasis, inducing specific dysbiosis coupled with distinct metabolic reprogramming, and that these jointly drive diarrheal pathogenesis.

To bridge this knowledge gap, we employed an integrative multi-omic approach, combining full-length 16S rRNA gene sequencing with untargeted liquid chromatography-mass spectrometry (LC-MS) metabolomics, to profile fecal samples from both healthy and diarrheal animals following a spontaneous diarrhea outbreak in a captive colony with exposure to visibly moldy corn. This pilot study aims to: (1) characterize the structural dysbiosis of gut microbiota; (2) identify key differential microbial taxa and metabolites associated with diarrheal pathogenesis; and (3) construct potential “microbe-metabolite” interaction networks that may drive the disease process. Our findings are expected to elucidate key microbial-metabolic mechanisms underlying diarrhea in this species, providing a foundational reference for intestinal health management, disease prevention, and the conservation management of *T. xanthipes* in captive breeding programs.

## 2. Results

### 2.1. Clinical Signs and Gross Pathological Lesions

**Figure 1:**
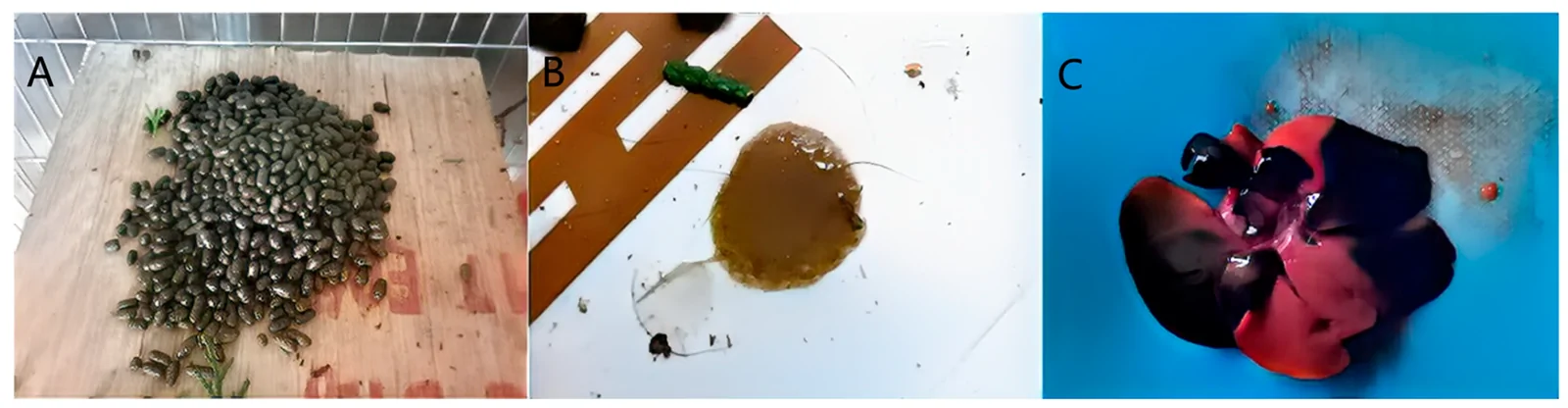
Clinical fecal morphology and representative hepatic gross lesions in complex-toothed flying squirrels (*Trogopterus xanthipes*).(**A**) Fecal morphology showing typical pellet shape; (**B**) Pale yellow-brown mucoid watery diarrhea.; (**C**) Postmortem view showing diffuse black discoloration and firm texture in affected liver lobes.

As shown in Figure 1, feces from healthy animals are compact and oval-shaped (Figure 1A), whereas diarrheal animals showed watery diarrhea characterized by pale yellow-brown, unformed, mucus-rich fluid feces with irregular spreading (Figure 1B), accompanied by prolonged lethargy, anorexia, and progressive emaciation. Postmortem examination reveals that the core affected areas appear black, predominantly involving the right lobe (anterior superior, anterior inferior, and posterior inferior segments), the caudate lobe, the quadrate lobe, and the left medial surface. The liver parenchyma is firm in consistency (Figure 1C). Field investigation showed that all affected animals had been fed with visibly moldy corn before the onset of illness.

### 2.2. Shifts in Gut Microbial Diversity and Compositional Architecture Associated with Diarrhea

**Figure 2:**
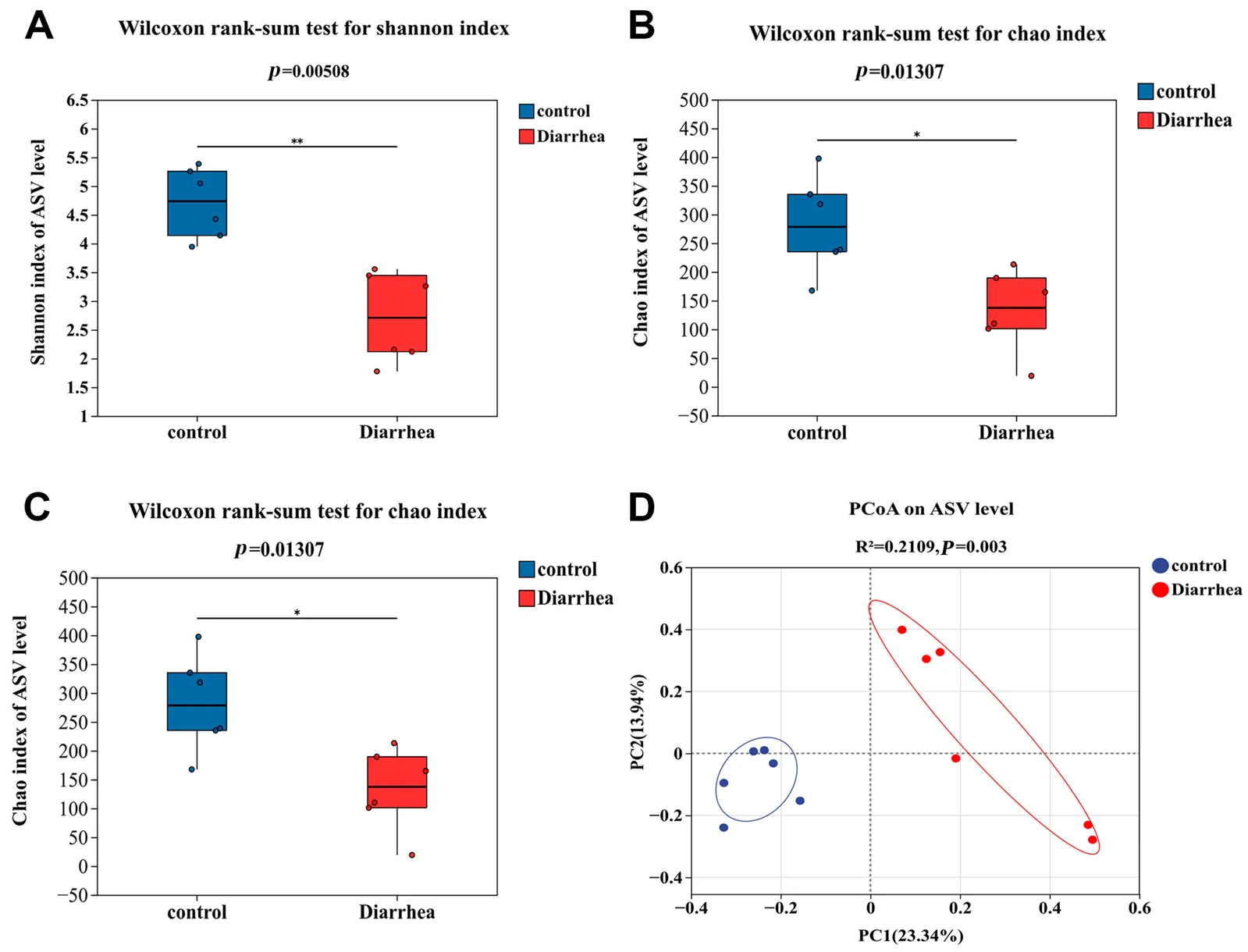
Gut microbiome diversity between the control and diarrhea groups at the ASV level. (**A**–**C**) α-diversity metric (ACE, Chao1, and Shannon) of the gut microbiome in the control and diarrhea groups. Statistical significance was determined by the Wilcoxon rank-sum test (*p* < 0.05); (**D**) PCoA plots based on Bray–Curtis dissimilarity at the ASV level. The percentages of variance explained by the first two principal coordinates (PC1 and PC2) are indicated. Ellipses depict 95% confidence intervals. Group separation was statistically significant as determined by PERMANOVA $R^{2}=0.2109,\;p=0.003$. Statistical significance: * *p* < 0.05, ** *p* < 0.01.

To assess the impact of diarrhea on gut microbial alpha diversity, we calculated the Abundance-based Coverage Estimator (ACE), Chao1, and Shannon indices at the amplicon sequence variants (ASV) level. Compared with the control group, the diarrhea group exhibited significantly lower community richness, as indicated by both ACE p=0.013 and Chao1 p=0.013 indices (Figure 2A,B). The Shannon index, which reflects community evenness and overall alpha diversity, was also significantly reduced in the diarrhea group (*p* = 0.005; Figure 2C). These results demonstrate that diarrhea is associated with marked reductions in both the richness and overall diversity of the gut microbiota. At the ASV level, we computed Bray–Curtis distances and visualized them using PCoA; the results revealed distinct clustering of microbial communities between the control and diarrhea groups (Figure 2D). Permutational multivariate analysis of variance (PERMANOVA) confirmed significant structural separation $R^{2}=0.211,\;p=0.003$, indicating substantial remodeling of the gut microbial structure was markedly altered during diarrhea.

### 2.3. Diarrhea-Driven Restructuring of Gut Microbiome and Identification of Differential Taxa

**Figure 3:**
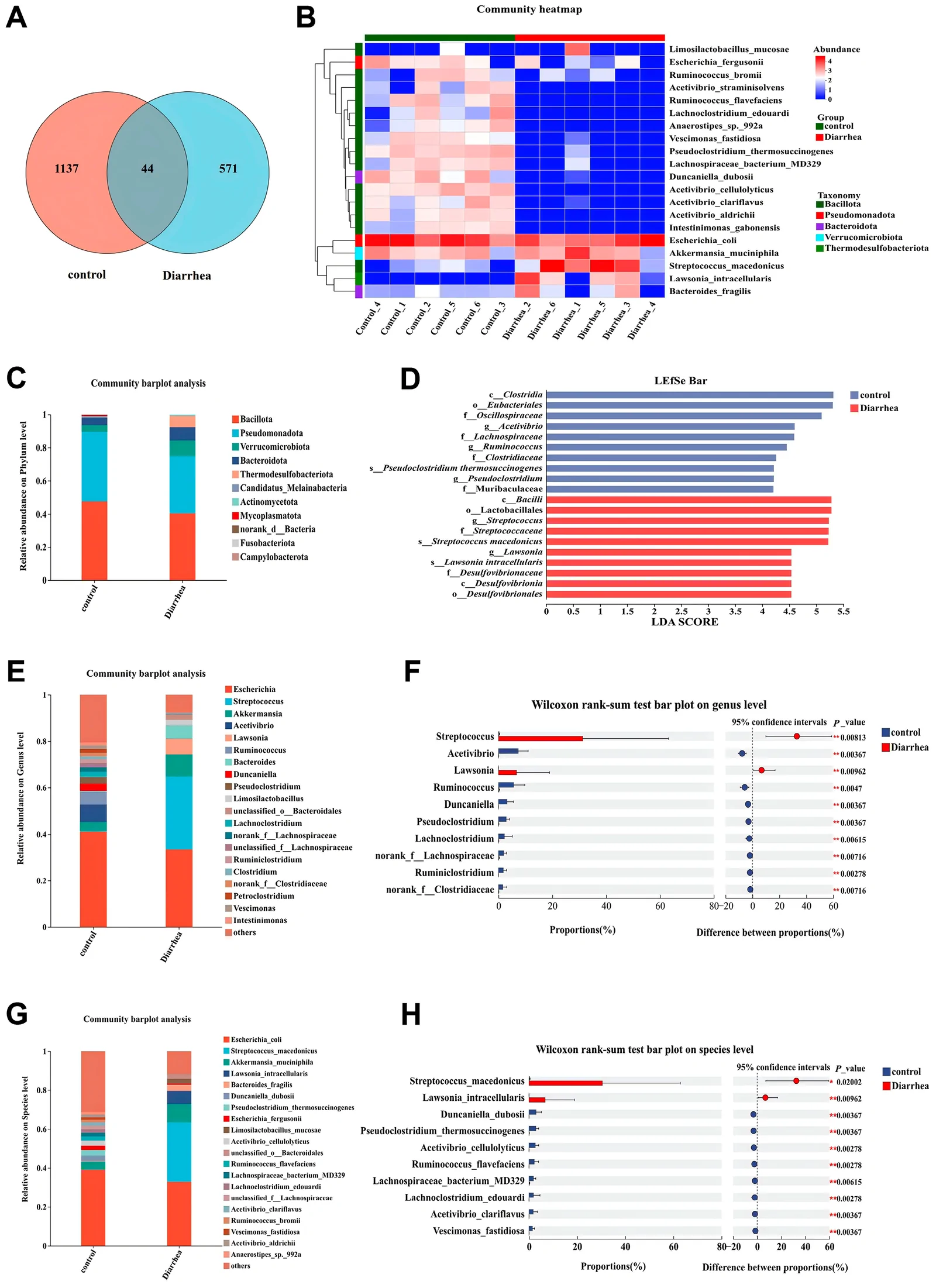
Gut microbiota composition and differences between control and diarrhea groups. (**A**) The Venn diagram displays the ASVs between control and diarrhea groups; 1137 were unique to control, 571 were unique to diarrhea and 44 were shared; (**B**) hierarchical clustering heatmap of microbial community structure based on relative abundance. Rows represent microbial taxa, columns represent individual samples, and color intensity indicates relative abundance; (**C**,**E**,**G**) bar plots illustrating the relative abundance of dominant microbial taxa at the phylum (**C**), class (**E**), and species (**G**) levels in control and diarrhea groups. Each bar represents a sample, and different colors correspond to distinct taxa; (**D**) LEfSe bar plot identifying taxa with significant biological differences between groups; (**F**,**H**) Wilcoxon rank-sum test bar plots validating significant differences in microbial relative abundance at the genus (**F**) and species (**H**) levels. Red represent diarrhea group; blue represent the control group (**D**,**F**,**H**). Statistical significance: * *p* < 0.05, ** *p* < 0.01.

The abundance data of 12 samples were a uniform depth of 14,076 sequences per sample, yielding a total of 1752 amplicon sequence variants (ASVs). The Venn diagram (Figure 3A) revealed distinct ASV composition between the control and diarrhea groups: 1137 ASVs were exclusively detected in the control group, 571 ASVs were unique to the diarrhea group, and only 44 ASVs were shared between the two groups, indicating substantial divergence in gut microbiota structure.

A hierarchical clustering heatmap (Figure 3B) based on microbial relative abundance further confirmed clear separation between control and diarrhea samples, with distinct clustering patterns for each group. Bar plots at the phylum (Figure 3C), class (Figure 3E), and species (Figure 3G) levels visualized the taxonomic composition of gut microbiota, showing prominent shifts in the proportional representation of dominant taxa across cohorts. Linear discriminant analysis effect size (LEfSe) was performed to identify taxa with significant biological differences between groups (Figure 3D). Taxa enriched in the control group included *Acetivibrio*, *Ruminococcus*, and *Pseudoclostridium* (*Pseudoclostridium thermosuccinogenes*), while taxa significantly enriched in the diarrhea group included *Streptococcus* (*Streptococcus macedonicus*), and *Lawsonia* (Lawsonia intracellularis), with LDA scores > 4.0 indicating strong effect sizes.

Wilcoxon rank-sum tests validated these differences at the genus (Figure 3F) and species (Figure 3H) levels. For genera, *Streptococcus* and *Lawsonia* showed significantly higher abundance in the diarrhea group (*p* < 0.01), whereas *Acetivibrio*, *Ruminococcus*, *Duncaniella*, *Pseudoclostridium*, *Lachnoclostridium* and *Ruminiclostridium* were enriched in the control group (*p* < 0.01). At the species scale, *Streptococcus macedonicus* and *Lawsonia intracellularis* were identified as the most prominent differential species in the diarrhea group (*p* < 0.05), consistent with LEfSe results.

### 2.4. Non-Targeted Metabolomics Reveals Distinct Fecal Metabolic Profiles Associated with Diarrhea

An untargeted metabolomics approach was employed to identify differences in key metabolites and metabolic pathways between the control group and the diarrhea group. The total ion chromatograms (TIC) of the metabolomics analysis are shown in Supplementary Figure S1. The relative standard deviation (RSD) of the quality control (QC) samples is presented in Supplementary Figure S2. These QC data indicate that data preprocessing effectively reduced technical variability, thereby meeting the quality control standards required for subsequent statistical analyses.

**Figure 4:**
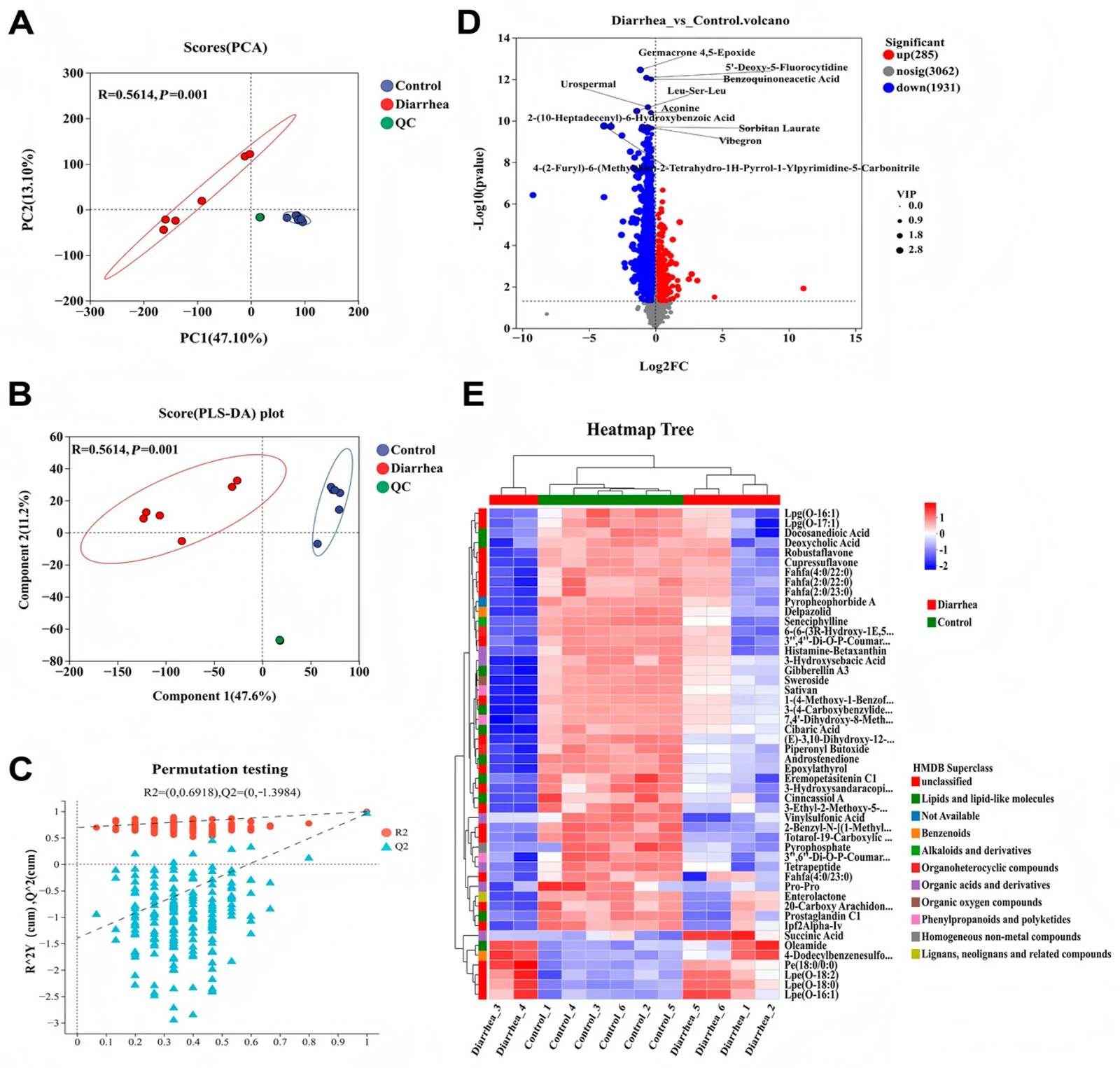
Multivariate statistical analysis of control and diarrhea samples. (**A**) PCA score plot showing the natural clustering of samples (control: blue, diarrhea: red, QC: green). PC1 and PC2 explain 47.10% and 13.10% of variance, respectively (R = 0.5614, *p* = 0.001); (**B**) PLS-DA score plot demonstrating clear separation between control and diarrhea groups. Samples are colored as in (**A**); (**C**) permutation test plot of the PLS-DA model (n = 200 permutations). The x-axis represents the permutation retention rate (the proportion of samples maintaining their original order relative to the Y variable). A data point with a retention rate of 1 corresponds to the R2 and Q2 values of the original model. The y-axis indicates the calculated values of R2 (red circles) and Q2 (blue triangles) from the permutation test. The two dashed lines represent the regression lines fitted for R2 and Q2, respectively; (**D**) volcano plot identifying deferentially abundant metabolites between diarrhea and control groups. Each point in the figure represents a specific metabolite, red: up-regulated, blue: down-regulated, gray: no significance; point size indicates VIP value; (**E**) heatmap of differential metabolite clustering. Heatmap displays the metabolic profiles of significantly altered metabolites in control and diarrheal groups. Rows denote metabolites (annotated with HMDB classifications), columns represent biological samples, and the red–blue color gradient reflects relative metabolite abundance red=high, blue=low. The dendrogram on the left shows the hierarchical clustering results of these 50 differentially expressed metabolites; different colors on the left represent different HMDB superclass classifications.

Unsupervised principal component analysis (PCA) showed a separation between the control group and the diarrhea group along the PC1 axis R=0.5614, p=0.001, with QC samples well clustered (Figure 4A). Partial Least Squares Discriminant Analysis (PLS-DA) was performed to further evaluate metabolic differences between the control and diarrhea groups. The PLS-DA score plot revealed distinct separation between the two groups (Figure 4B). A permutation test (n = 200 permutations) confirmed the robustness and reliability of the PLS-DA model (Figure 4C). Detailed model parameters for the PLS-DA components are provided in Supplementary Table S1.

A total of 2216 differential metabolites were identified between the two groups. with detailed information provided in Supplementary Table S2. Among them, 285 were up-regulated and 1931 were down-regulated. The volcano plot visually depicts the distribution of these differential metabolites (Figure 4D). Differential metabolites were primarily classified into categories including lipids and lipid-like molecules, phenylpropanoids and polyketides, organic acids and derivatives, organoheterocyclic compounds, and benzenoids. Following normalization of metabolite expression levels, a heatmap of the top 50 most differentially expressed metabolites (Figure 4E).

### 2.5. Metabolic Pathway and Functional Enrichment Analysis of Differential Metabolites

**Figure 5:**
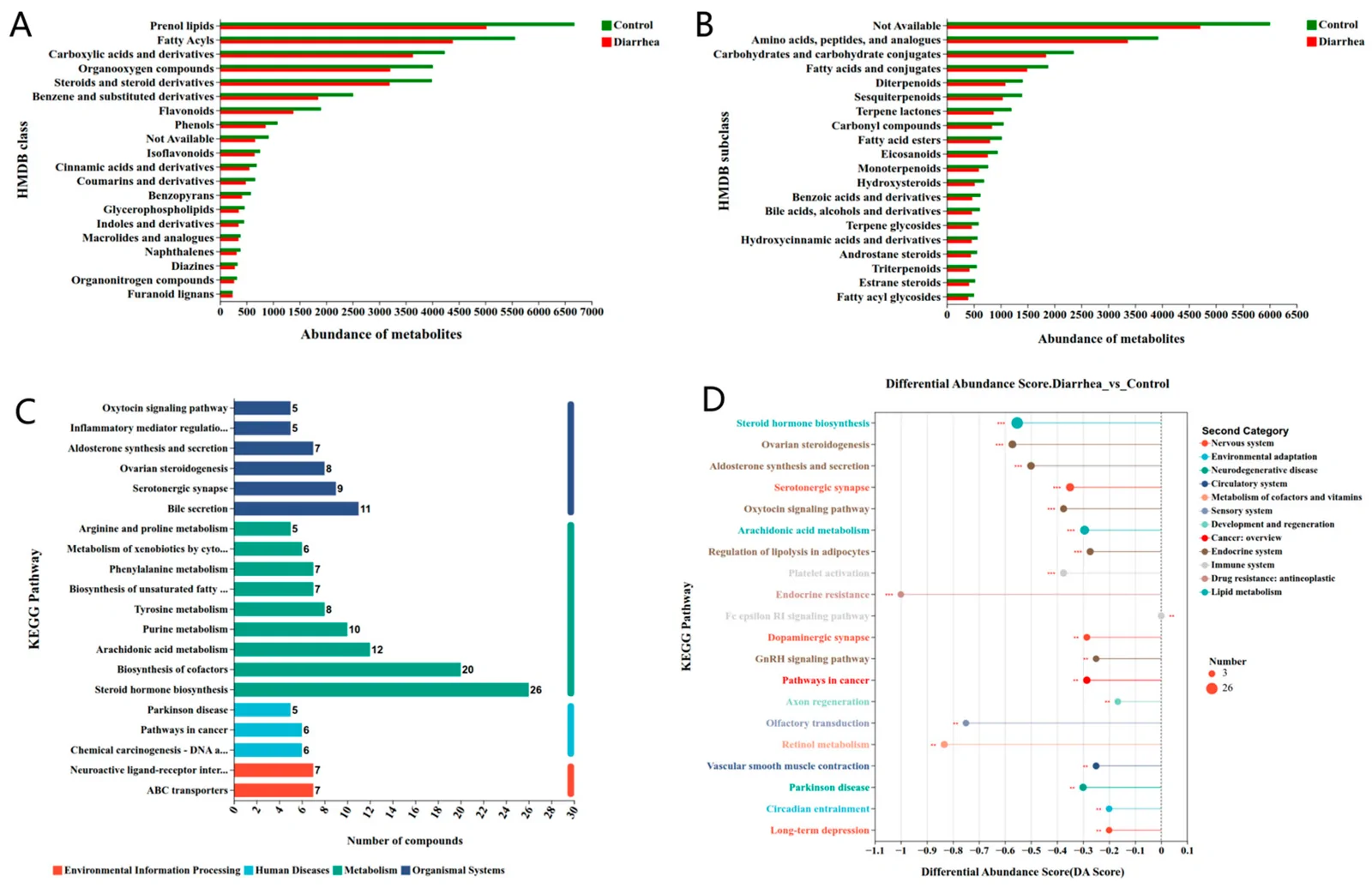
Comprehensive metabolomic and pathway-level differences between diarrhea and control groups. (**A**) HMDB class-level metabolite abundance. control (green) and diarrhea (red) groups; (**B**) HMDB subclass-level metabolite abundance. control (green) and diarrhea (red) groups; (**C**) enrichment analysis of differential metabolites across KEGG pathway. The y- axis denotes the secondary-level of the KEGG metabolic pathway classifications, while the x-axis shows the number of compounds assigned to the respective pathway. The color of the bars on the right indicates different metabolic pathway categories; (**D**) differential abundance score (DA Score) for KEGG pathways (diarrhea vs. control). The x-axis represents the DA Score (ranging from −1 to 1), where negative values indicate pathway down-regulation in the diarrhea group, and positive values indicate up-regulation. The length of the horizontal line reflects the absolute value of the DA Score. Dot size corresponds to the number of differential metabolites annotated to each pathway, and colors represent secondary functional categories. **: corrected *p* < 0.01, ***: corrected *p* < 0.001.

Metabolites were mapped to 14 superclasses in the HMDB database. At the class level (Figure 5A), the diarrhea group had lower abundances of major metabolite classes such as prenol lipids and fatty acyls compared with the control group. At the subclass level (Figure 5B), the abundances of subclasses including amino acids, peptides, and analogs, as well as carbohydrates and carbohydrate conjugates, were reduced in the diarrhea group.

To explore the metabolic mechanisms underlying diarrhea, KEGG pathway enrichment and differential abundance analysis on metabolites identified between the diarrhea and control groups. A total of 53 differential metabolic pathways were enriched (Supplementary Table S3), with the top 20 ranked by *p*-value illustrated in Figure 5C. The five pathways with the largest numbers of differential metabolites were steroid hormone biosynthesis (n=26), biosynthesis of cofactors (n=20), arachidonic acid metabolism (n=12), bile secretion (n=11) and purine metabolism (n=10). These pathways fall under metabolism and organismal systems.

Pathway activity trends were further assessed using differential abundance (DA) scores, with the top 20 most significantly altered pathways shown in Figure 5D. Among these, the steroid hormone biosynthesis pathway showed the highest enrichment level. All pathways exhibited a DA Score ≤ 0, indicating a general suppression of metabolic pathway activity due to diarrhea, with down-regulated pathways were predominantly associated with lipid metabolism, endocrine system, and nervous system.

**Figure 6:**
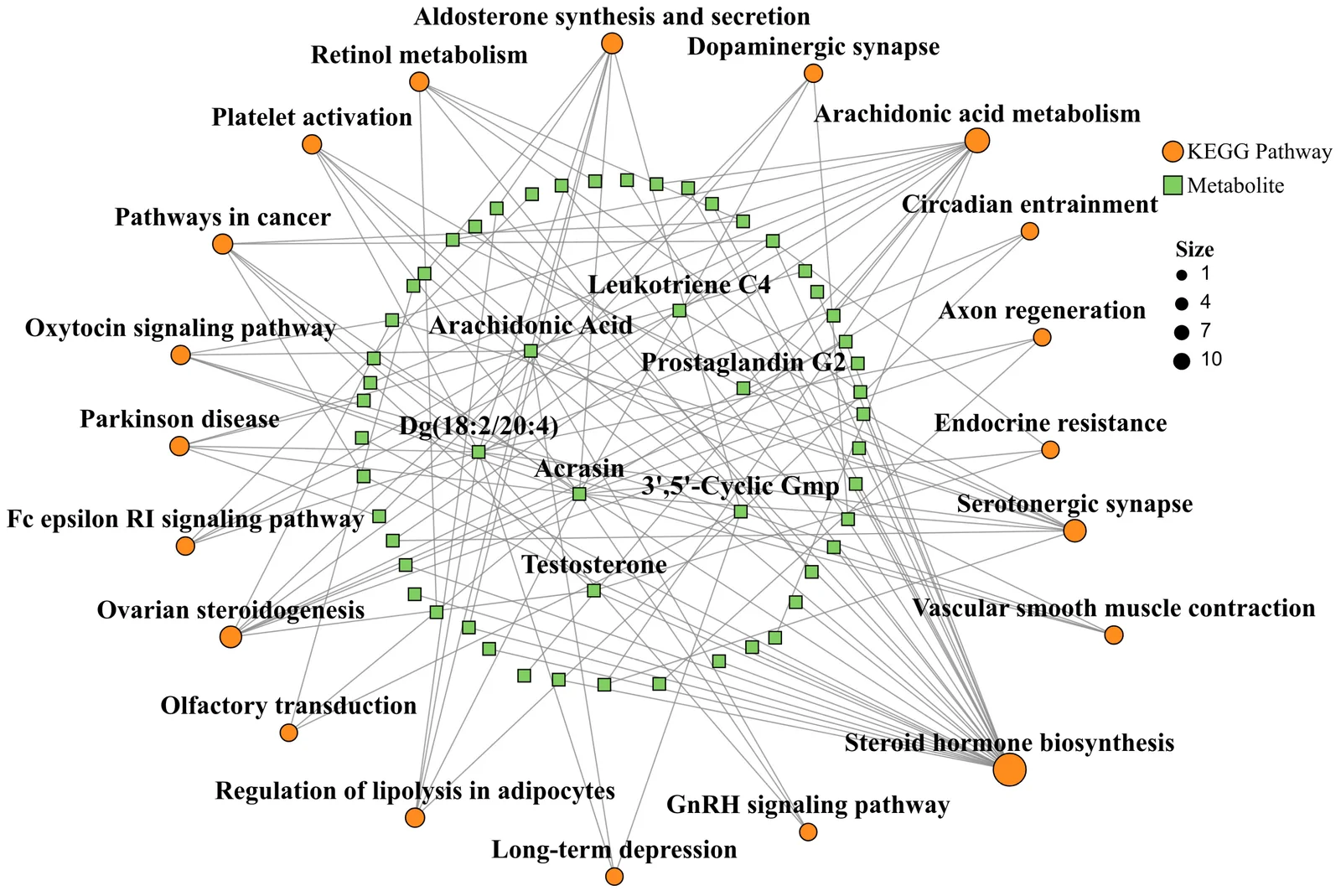
Differential metabolite-KEGG pathway association network diagram. Green squares denote metabolites; orange circles represent KEGG pathways, with circle size proportional to the number of associated metabolites (larger circles indicate more metabolites in the pathway).

Based on these findings, to further elucidate the regulatory interactions between metabolites and metabolic pathways during diarrhea, we constructed a metabolite–pathway interaction network using the top 20 key pathways (Figure 6). The integrated analysis indicates that steroid hormone biosynthesis, arachidonic acid metabolism, ovarian steroidogenesis, and serotonergic synaptic signaling are the core pathways of metabolic disorders in the diarrheal group. Acrasin and arachidonic acid, as key metabolites, serve as core nodes and are closely associated with 15 key pathways including steroid hormone biosynthesis. Among them, metabolites such as 3′,5′-Cyclic GMP, DG(O-18:3/2:0), prostaglandin F2α, androstenedione, testosterone, leukotriene C4, prostaglandin G2, and corticosterone are involved in three or more KEGG pathways, suggesting that they are key hub molecules in the diarrheal metabolic regulation network.

### 2.6. Overall Concordance Between Microbiome and Metabolome and the Key Microbiota-Metabolite Association Network

**Figure 7:**
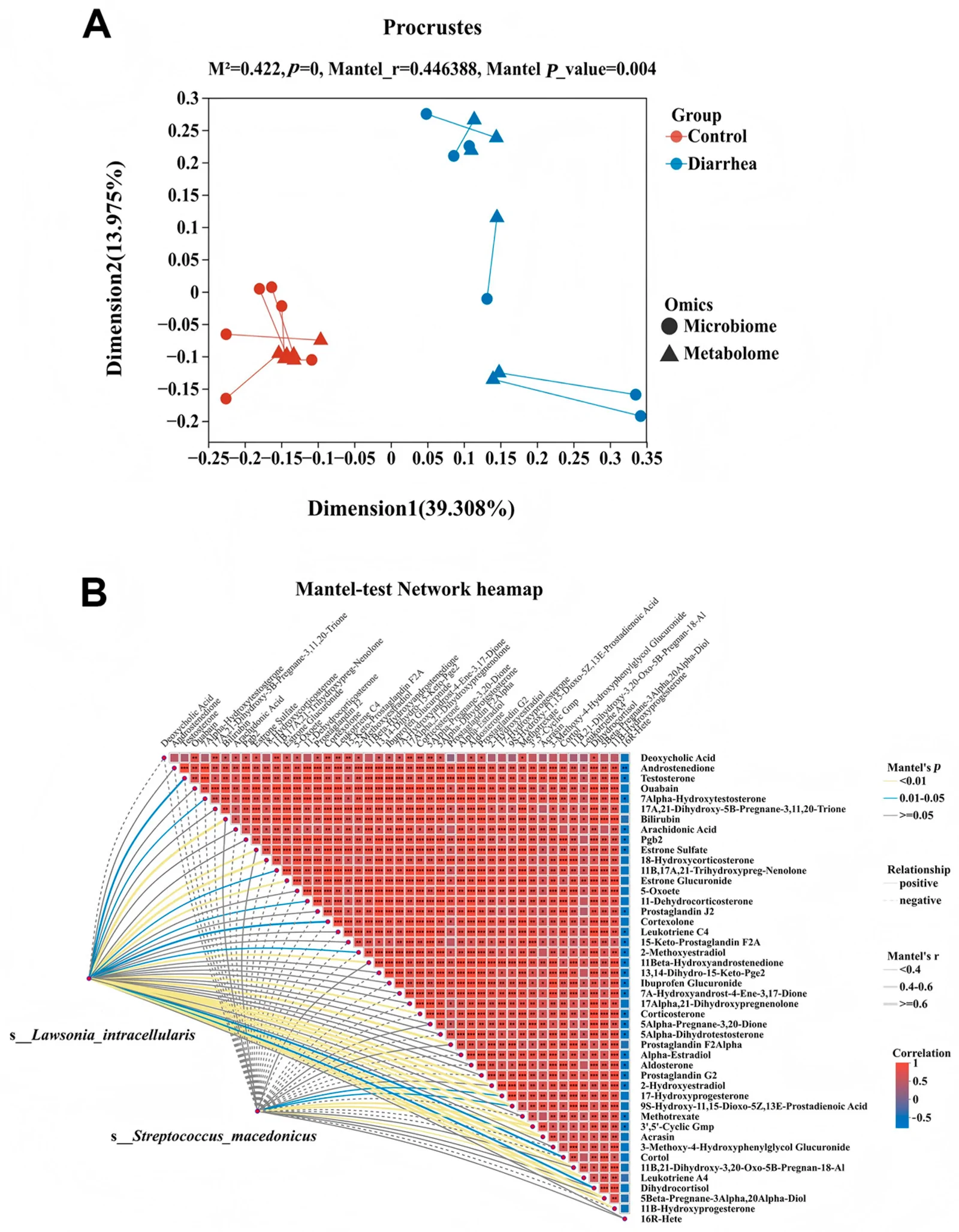
Associations between gut microbiome and metabolome in diarrhea-affected vs. control *Trogopterus xanthipes*. (**A**) Procrustes analysis integrates microbiome (circles) and metabolome (triangles) profiles, with lines linking paired omics data for each sample. Procrustes M2 = 0.422 (*p* < 0.001) and Mantel’s (r = 0.446 *p* = 0.004) indicating significant omics concordance and group separation. (**B**) Mantel-test-based network heatmap showing correlations between key gut microbiota and metabolites in *Trogopterus xanthipes* with diarrhea. Lines connect microbiota and metabolites, with line thickness scaled according to the absolute Mantel’s r value (thicker line = larger Mantel’s r). The direction of each microbiota-metabolite correlation (positive or negative) is indicated by the line’s “relationship” label (positive/negative). The heatmap cells display Pearson correlation for a given metabolite pair. Asterisks within the heatmap cells indicates statistical significance, with the following *p*-value criteria: * for 0.01 < *p* ≤ 0.05, ** for 0.001 < *p* ≤ 0.01, and *** for *p* ≤ 0.001.

Procrustes analysis was performed to assess the overall concordance between the two omics profiles between the control and diarrhea groups (Figure 7A). The analysis revealed a significant congruence between the microbiome and metabolome datasets (Procrustes M^2^ = 0.422, *p* < 0.001). A complementary Mantel test further confirmed a significant positive correlation between microbiome and metabolome dissimilarity matrices (Mantel’s r = 0.446, *p* = 0.004).

A Mantel-test correlation network was constructed based on Spearman correlation analysis to investigate associations between differential metabolites derived from the top three KEGG pathways (ranked by the number of annotated metabolites) and key bacterial taxa (Figure 7B). The results showed that diarrhea-associated key species (*L. intracellularis* and *S. macedonicus*) exhibited distinct correlation patterns with these metabolites. *L. intracellularis* showed significant positive correlations with multiple metabolites (*p* < 0.01, r ≥ 0.4), with particularly strong associations for testosterone (r=0.523), bilirubin (r=0.698), 18-Hydroxycorticosterone (r = 0.705), and prostaglandin F2α r=0.660. In contrast, *S. macedonicus* showed significant correlations (*p* < 0.05) with only five specific metabolites: 17-hydroxyprogesterone (r=0.345), 3′,5′-cyclic Gmp (r=0.412), acrasin (r=0.478), 9S-hydroxy-11,15-dioxo-5Z,13E-prostadienoic acid (r = 0.314), and leukotriene A4 (r=0.399). Notably, both bacterial species exhibited positive correlations with acrasin and leukotriene A4.

## 3. Discussion

The gastrointestinal tract is not merely a site for mycotoxin absorption but a critical interface where host-microbiota interactions dictate toxicological outcomes [21]. In this study, we preliminarily revealed synergistic alterations in the gut microbiome and metabolome of *Trogopterus xanthipes* with diarrhea, although the causative agents remain unidentified. Our findings reveal correlational association between this diarrheal state and a complex pathophysiological condition characterized by gut dysbiosis along with host metabolic disturbances.

Our data demonstrate that diarrhea animals exhibit marked gut ecological instability, characterized by decreased α-diversity and distinct β-diversity clustering. These findings resemble patterns of mycotoxin-associated dysbiosis reported in other monogastric animals. For instance, deoxynivalenol (DON) exposure has been reported to induce comparable decreases in α-diversity and shifts in microbial composition in broilers, pigs, and mice [22,23]. Similarly, combined challenges with multiple mycotoxins, including aflatoxin B1(AFB_1_), ochratoxin A(OTA), and zearalenone (ZEN), produce comparable effects [24]. However, given the lack of mycotoxin quantification in our feed samples, these parallels remain purely speculative and serve only as hypothesis-generating observations. We cannot attribute these microbial changes specifically to toxin exposure; alternative drivers, including enteric pathogens, may also contribute to the observed dysbiosis.

At the compositional level, the diarrhea group exhibited two key changes: enrichment of opportunistic pathogens (e.g., *Streptococcus* and *Lawsonia intracellularis*) and depletion of beneficial bacteria (members of the class Clostridia, such as *Ruminococcus* and *Lachnoclostridium*). *Streptococcus* has been reported as opportunistic pathogens in the swine [25], potentially exacerbating intestinal injury through inflammation pathways. A significant increase in *Streptococcus* abundance was also recently observed in a calf diarrhea model induced by enterotoxigenic *Escherichia coli* K99 [26]. Moreover, the detection of *L. intracellularis* in *T. xanthipes* extends the known host range of this pathogen. *L. intracellularis* is associated with proliferative enteropathy in pigs [27] and horses [28], and was recently documented in a broader range of hosts, including chickens [29], rabbits [30], and various wild rodents and feral cats [31,32,33]. Given its ability to induce epithelial hyperplasia [34], we hypothesize that *L. intracellularis* may contribute to diarrhea by compromising intestinal mucosal integrity and nutrient absorption. Concurrently, there was a reduction in beneficial members of the class Clostridia, which are core fibrolytic bacteria essential for carbohydrate fermentation and energy harvest [35,36]. Their depletion may impair the host’s digestive capacity, creating a vicious cycle of malnutrition and diarrhea. This pattern is consistent with observations in calf and piglet diarrhea [37,38].

Although the observed clinical signs and metabolic signatures show some concordance with documented mycotoxin exposure, such as AFB_1_-induced comprehensive inhibition of steroid hormone synthesis, arachidonic acid metabolism, and bile acid dynamics [39], we cannot definitively confirm causality or identify the specific toxins involved. This cross-species phenotype aligns with the intestinal phenotypes reported following exposure to major mycotoxins, including AFB_1_, DON, OTA and ZEN. These mycotoxins disrupt intestinal homeostasis through multidimensional mechanisms affecting the physical barrier (tight junctions/villus structure), chemical barrier (mucus/secretion), immune barrier, and microbiota interactions [40].

Nevertheless, it is instructive to compare our metabolic findings with established mycotoxin pathophysiology, as this may inform future targeted analyses. At the level of steroid hormones, the marked reduction of key metabolites including corticosterone and testosterone indicates impaired steroid synthesis in intestinal epithelial cells. For AFB_1_, it has been shown to suppress key steroidogenic enzymes, such as StAR and HSD17B3 via the ROS/AMPK signaling pathway, thereby obstructing testosterone synthesis [41,42]. Notably, corticosterone, as an endogenous glucocorticoid, exerts pivotal barrier-protective effects in the gut; its depletion may release the “brakes” on intestinal secretion and inflammatory injury, exacerbating pathological damage [43]. Chronic mucosal inflammation further inhibits 11β-hydroxysteroid dehydrogenase type 2 11β−HSD2, the key enzyme responsible for converting inactive dehydrocorticosterone into active corticosterone, thus potentially establishing a vicious cycle that prolongs inflammation [44,45]. In arachidonic acid metabolism, the downregulation of prostaglandin F_2_α (PGF_2_α) and leukotriene C_4_ (LTC_4_) carries important pathological implications. While PGF_2_α regulates smooth muscle contraction, its metabolite (e.g., 11β-PGF_2_α) may promote epithelial repair [46]; therefore, PGF_2_α depletion likely represents a contributing factor to barrier dysfunction. More critically, the reduction in LTC_4_-an upstream lipid mediator essential for initiating type 2 immunity-suggests possible impairment of the epithelial-derived cysLT-mediated “alarmin” signaling axis. The absence of LTC_4_ attenuates the activation of group 2 innate lymphoid cells (ILC2s) and dendritic cells, subsequently inhibiting the recruitment and infiltration of eosinophils to sites of injury [47]. The concurrent suppression of these two arachidonic acid metabolites may compromise the intestinal capacity to eliminate pathogenic bacteria, facilitating the colonization of *L. intracellularis* and *Streptococcus*.

Additionally, bile acid metabolism disorder has been identified as a key pathogenic alteration. This finding closely resembles the dysregulation pattern of the “microbiota–bile acid-host axis” observed in calf diarrhea induced by enterotoxigenic *Escherichia coli* K99 [26], Moreover, another study has indicated that DON toxin may further disrupt enterohepatic circulation and intestinal barrier homeostasis by inhibiting bile acid transporters (ASBT/IBABP/OSTα) or altering the microbiota’s capacity to convert bile acids [48]. Notably, the above-described metabolic changes are consistent with the expression regulation of upstream key enzymes. It has been demonstrated that ochratoxin A (OTA) significantly downregulates the gene expression of cyclooxygenase-2 (COX-2) and 5-lipoxygenase (5-LOX) in intestinal epithelial cells [49]. Given that COX-2 is the rate-limiting enzyme for PGF_2_α synthesis and 5-LOX is the key initiating enzyme for LTC_4_ synthesis, this finding provides a plausible enzymatic explanation for the reduced levels of PGF_2_α and LTC_4_ observed in our metabolomic analysis. In summary, we infer that the toxin mixture in moldy corn may serve as the initial driving force triggering subsequent gut dysbiosis and metabolic suppression, rather than being a mere epiphenomenon of diarrhea.

Based on the microbe–metabolite association network derived from Mantel tests, a speculative pathological model can be proposed, though it requires cautious interpretation. Additionally, toxin exposure has been reported to induce intestinal epithelial cell death [50], thereby disrupting microbiota–host interactions. Within this context, *L. intracellularis*, which was significantly enriched in the diarrhea group, exhibited extensive positive correlations with steroid hormones and arachidonic acid metabolites, suggesting that it directly drives the pathological process by hijacking the host endocrine and inflammatory signaling. In contrast, *Streptococcus macedonicus* showed significant but generally weaker positive correlations with only five specific metabolites (r from 0.314 to 0.478), implying that it may represent a commensal colonizer or a passive responder to environmental disturbances. This comparison indicates that different bacterial taxa may drive diarrhea through distinct biochemical pathways, an ecological logic consistent with the “microbiota–metabolite–growth performance” association models constructed by Xue et al. [51] and Zhuang et al. [52] in calves. Notably, certain metabolites (e.g., acrasin and leukotriene A4) exhibited concordant positive correlations with both bacterial species, suggesting that different taxa may influence host diarrhea-associated phenotypes via distinct metabolic routes. Furthermore, the widespread strong positive inter-correlations among the metabolites suggest functional coupling between steroid and arachidonic acid pathways, collectively shifting the host metabolic profile from normal homeostatic fermentation toward a pathological state characterized by gut dysbiosis, inflammatory activation, and stress injury.

It is tempting to speculate that mycotoxin contaminants—if present in the moldy corn—could act as an upstream perturbing factor in this pathological ecosystem. However, this hypothesis does not exclude the alternative scenario that the detected pathogens are the primary drivers of diarrhea, with the observed metabolic changes being a consequence rather than a cause of the infectious process. Our data collectively suggest a complex pathological network in which host metabolism, microbial ecology, and potential toxic exposures are intertwined, but the causal architecture remains unresolved.

Finally, species-specific metabolism in context deserves attention. As a rodent species that exclusively feeds on *Platycladus orientalis*, the *Trogopterus xanthipes* produces feces (known as Wulingzhi) that are not merely excreta but also unique chemical carriers of host–microbial co-metabolism. Modern studies have revealed that Wulingzhi contains various bioactive components, including terpenoids, flavonoids, and phenolic acids, and exhibits anti-inflammatory and analgesic effects [53,54]. In organisms, steroid hormones are derived from the triterpene precursor squalene through cyclization and subsequent multi-step modifications (including side-chain cleavage, oxidation, and reduction), with both classes of compounds sharing the mevalonate (MVA) biosynthetic pathway [55]. In this study, the levels of both steroid hormones and terpenoids were reduced in the diarrheal feces (Supplementary Table S2), suggesting possible impairment of upstream MVA flux or metabolic disruption. This finding indicates a direct biochemical coupling between the host’s endocrine health status and the chemical composition of Wulingzhi. Disruption of hormone levels may not only compromise the host’s stress adaptation and endocrine homeostasis but also alter its metabolite profile, potentially affecting the raw material quality of Wulingzhi. This interplay between pathophysiological processes and the quality of natural medicinal resources underscores the importance of considering host health status and metabolic regulatory mechanisms in the conservation and utilization of animal-derived medicinal materials such as Wulingzhi.

### Limitations

Several limitations should be acknowledged. First and foremost, we did not perform mycotoxin quantification, or identification from the feed samples. Despite visual observations of moldiness (black appearance, musty odor) and clinical signs suggestive of toxic exposure, the absence of analytical chemistry data precludes any definitive attribution of the observed phenotypes to specific mycotoxins. Consequently, our findings serve as correlational evidence and hypothesis-generating observations, rather than causal proof. Second, the sample size was relatively modest (n = 6 per group), which may reduce statistical power for detecting subtle differences. Third, we did not perform qPCR for absolute quantification of specific pathogens, nor did we preserve sufficient sample aliquots for such assays; future studies incorporating qPCR are needed to validate the pathogen loads identified here. Fourth, given the observational design, we cannot infer causality-whether the observed microbial and metabolomic alterations are drivers or consequences of diarrhea remains to be determined through experimental or longitudinal investigations.

## 4. Conclusions

In summary, we provide a multi-omic characterization of diarrhea in *Trogopterus xanthipes*. We demonstrate that diarrhea is associated with gut dysbiosis (reduced diversity, loss of fiber-degrading bacteria, and expansion of potential pathobionts such as *Lawsonia intracellularis* and *Streptococcus*) and altered metabolism of lipid, steroid, and bile acid. Notably, these metabolic disruptions show marked similarities to the intestinal toxic phenotypes induced by common mycotoxins (e.g., AFB_1_, DON, OTA) in other animal models, although we did not detect these toxins directly. Our findings suggest that exposure to moldy corn is associated with a coordinated set of microbe-host metabolic alterations that may drive diarrheal pathogenesis. We hypothesize that mycotoxin contaminants—if present in the moldy corn—could be a potential key factor mediating the gut pathological changes observed in this study. However, we emphasize that this remains a speculative model; the detected enteropathogens represent an equally compelling alternative primary cause.

## 5. Materials and Methods

### 5.1. Experimental Animals and Fecal Sample Collection

Complex-toothed flying squirrels were obtained from a captive breeding farm in Luonan County, Shangluo City, Shaanxi Province, China. Examination of the stored corn feed showed severe mold growth, as evidenced by black discoloration and a musty smell. Affected animals exhibited clinical symptoms including lethargy, anorexia, progressive watery diarrhea, emaciation, and death.

Twelve fecal samples were collected from the same farm, including 6 from animals with diarrhea (diarrhea group) and 6 from healthy animals (control group). The diagnostic criteria for diarrhea disease were as follows: (i) unformed feces, (ii) continuous diarrhea lasting more than three days. Fecal samples were collected by inserting a sterile swab approximately 2–3 cm into the anus, gently rotating the swab, and then immediately placing it into a sterile tube. For the control group, healthy animals from the same farm with similar rearing cycles and housing conditions but without any exposure to moldy corn were selected, and fecal samples were collected using the identical swabbing method. All samples were maintained at −80 °C until required for experiments.

### 5.2. Full-Length 16S rRNA Sequencing and Bioinformatics Analysis

Total microbial genomic DNA was extracted from the 12 samples using the FastPure Stool DNA Isolation Kit (MJYH, Shanghai, China). The full-length 16S rRNA gene was amplified with barcoded primers 27F (5′-AGRGTTYGATYMTGGCTCAG-3′) and 1492R (5′-RGYTACCTTGTTACGACTT-3′) [56], and PCR was conducted under the following conditions: 95 °C for 3 min, 27 cycles of 95 °C for 30 s, 60 °C for 30 s and 72 °C for 45 s, followed by a final extension at 72 °C for 10 min. Amplicons were purified using AMPure^®^ PB beads (Pacific Biosciences, Menlo Park, CA, USA) and quantified with Qubit 4.0 (Thermo Fisher Scientific, Waltham, MA, USA). SMRTbell libraries were sequenced on a PacBio Sequel IIe system (Pacific Biosciences, Menlo Park, CA, USA) and HiFi reads (1000–1800 bp) were generated using SMRT Link v11.0.

The bioinformatics pipeline was identical to that described in Chen et al. [57]. Briefly, DADA2 [58] within QIIME2 [59] (v 2020.2) was used to denoise optimized reads and generate ASVs. After rarefaction 6000 sequences (average Good’s coverage: 97.90%), taxonomic assignment was performed using the SILVA e (v138) database and the Naive Bayes classifier in Qiime2. Alpha diversity (observed ASVs, Chao1, Shannon and Good’s coverage) was calculated with Mothur v1.30.1 [60].

Beta diversity was assessed via PCoA based on Bray–Curtis distances using Vegan v2.5-3., with treatment effects tested by PERMANOVA, the linear discriminant analysis (LDA) effect size (LEfSe) [61] was employed to identify the taxa (phylum to species) with significant differential abundance (LDA score > 4.0, *p* < 0.05). For gut microbiota analysis, we rely on the Majorbio Cloud platform [62].

### 5.3. Untargeted Fecal Metabolomics

Fecal metabolites were extracted and analyzed via untargeted LC-MS/MS (Majorbio Biotech, Shanghai, China). Briefly, fecal samples (100 mg) were mixed with 800 μL of methanol/water (4:1, *v*:*v*) containing 0.02 mg/mL L-2-chlorophenylalanine as an internal standard. The subsequent extraction procedures were carried out as described previously [63]. Chromatographic separation was performed on an ACQUITY HSS T3 column (100 mm × 2.1 mm i.d., 1.8 μm; Waters, Milford, MA, USA) using a UHPLC-Q Exactive HF-X system. The mobile phases, flow rate, column temperature, MS conditions (ESI positive/negative modes, source temperature 400 °C, ion-spray voltage −2800 V/3500 V, data-dependent acquisition over m/z 70–1050). Raw data was performed by Progenesis QI v3.0 (Waters Corporation, Milford, MA, USA) software. Metabolites identification was performed by searching against the HMDB, Metlin, and an in-house database (Majorbio). For statistical analysis, PCA and OPLS-DA were conducted using the R package ropls (v1.6.2). Metabolites with VIP > 1 and “*p*” < 0.05 (Student’s *t*-test) were considered significantly different. Pathway enrichment analysis was carried out using KEGG and HMDB databases.

### 5.4. Integrated Microbiome–Metabolome Analysis

Spearman correlation was used to assess pairwise relationships between key bacterial species and differential metabolites. Procrustes analysis and Mantel test evaluated the concordance between two datasets. Correlation networks and Mantel-test network heatmaps were constructed to visualize significant associations.

## Data Availability

Full-length raw reads were deposited to the National Center for Biotechnology Information (NCBI) Sequence Read Archive (SRA) database (Accession Number: PRJNA1475744). The metabolomics data are deposited in the NGDC OMIX database (https://ngdc.cncb.ac.cn/omix) under accession number OMIX017553 (accessed on 8 June 2026).

## Supplementary Materials

The following supporting information can be downloaded at: https://www.mdpi.com/article/10.3390/toxins18080340/s1, Figure S1: Total Ion Chromatography; Figure S2: Cumulative RSD Distribution Plot of QC Samples; Supplementary Table S1: Parameters of the PLS-DA model; Supplementary Table S2: Statistics and database annotations of differential metabolites; Supplementary Table S3: KEGG differential metabolic pathways.

## Abbreviations

The following abbreviations are used in this manuscript:

ASV	amplicon sequence variants
ACE	Abundance-based Coverage Estimator
LEfSe	Linear discriminant analysis effect size
PLS-DA	Partial Least Squares Discriminant Analysis
PCA	principal component analysis
DON	deoxynivalenol
AFB1	aflatoxin B1
OTA	ochratoxin A
ZEN	zearalenone

## References

[1] Yang N.Y., Tao W.W., Zhu M., Duan J.A., Jiang J.G. (2009). Two new isopimarane diterpenes from the feces of *Trogopterus xanthipes*. Fitoterapia.

[2] Zhao J.C., Wang Y.H., Weng Q.Q. (2020). Herbal Textual Research on Faeces Trogopterori in Chinese Classical Prescriptions. Mod. Chin. Med..

[3] Yang N.Y., Tao W.W., Duan J.A. (2010). Antithrombotic flavonoids from the faeces of *Trogopterus xanthipes*. Nat. Prod. Res..

[4] Zhao J., Zhu H.J., Zhou X.J., Yang T.H., Wang Y.Y., Su J., Li Y., Cheng Y.X. (2010). Diterpenoids from the feces of *Trogopterus xanthipes*. J. Nat. Prod..

[5] Liu P.Y., Cheng A.C., Huang S.W., Lu H.P., Oshida T., Liu W., Yu H.-T. (2020). Body-size Scaling is Related to Gut Microbial Diversity, Metabolism and Dietary Niche of Arboreal Folivorous Flying Squirrels. Sci. Rep..

[6] Ma Y. (2024). The Role of Gut Microbiota in Dietary Transition of Juvenile *Trogopterus xanthipes*. Master’s Thesis.

[7] Nicholson J.K., Holmes E., Kinross J., Burcelin R., Gibson G., Jia W., Pettersson S. (2012). Host-Gut Microbiota Metabolic Interactions. Science.

[8] Li N., Guo X. (2026). The gut microbiota and host immunity synergistically orchestrate colonization resistance. Gut Microbes.

[9] Valencia S., Zuluaga M., Pérez M.C.F., Quintero K.F.M., Cortés M.S.C., Robledo S. (2025). Human Gut Microbiome: A Connecting Organ Between Nutrition, Metabolism, and Health. Int. J. Mol. Sci..

[10] Wang Q., Lu Q., Tang Y., Li Q., Gao P., Pang S., Zhang W., Nie C., Niu J., Ma X. (2025). Integrated 16S rDNA-Seq and metabolomics reveal seasonal dynamics of gut microbial–SCFA–immune crosstalk in diarrheic calves. Front. Vet. Sci..

[11] Zhang Y., Ma Y., Qi Y. (2025). Potential relationship between gut microbiota and animal diarrhea: A systematic review. Front. Microbiol..

[12] Xie F., Zhou M., Li X., Li S., Ren M., Wang C. (2024). Macrogenomic and Metabolomic Analyses Reveal Mechanisms of Gut Microbiota and Microbial Metabolites in Diarrhea of Weaned Piglets. Animals.

[13] Abdolmaleky H.M., Zhou J.R. (2024). Gut Microbiota Dysbiosis, Oxidative Stress, Inflammation, and Epigenetic Alterations in Metabolic Diseases. Antioxidants.

[14] Zhang C.Y., Peng X.X., Wu Y., Peng M.J., Liu T.H., Tan Z.J. (2024). Intestinal mucosal microbiota mediate amino acid metabolism involved in the gastrointestinal adaptability to cold and humid environmental stress in mice. Microb. Cell Factories.

[15] Hu X.S., Jiang D., Li T., Xu Z., Liu J.G., Liu C.S. (2019). Diversity of microbiota composition of traditional Chinese medicine of Faeces Trogopterori from Shangluo, Shanxi province by sequence of the bacterial 16S rRNA gene. Acta Pharm. Sin..

[16] Zhu S., Zou M., Wu Q., Zou Y., Tan T., Huang Z., Gong Z., Luo H., Dong X. (2026). The Gut-Liver Axis in Metabolic Dysfunction-Associated Steatotic Liver Disease: From Mechanistic Insights to Precision Therapeutics. FASEB J..

[17] Lan C., Ren W., Wu A., Yu B., He J., Luo Y., Chen D. (2025). Prevotella copri alleviates diarrhea in weaning piglets through gut microbiota modulation and arachidonic acid–AHR–NRF2 pathway activation. J. Anim. Sci. Biotechnol..

[18] Shi Z., Wang Y., Yan X., Ma X., Duan A., Hassan F.U., Wang W., Deng T. (2023). Metagenomic and metabolomic analyses reveal the role of gut microbiome-associated metabolites in diarrhea calves. mSystems.

[19] Zhan K., Zheng H., Li J.Q., Wu H.M., Qin S.M., Luo L., Huang S. (2020). Gut Microbiota-Bile Acid Crosstalk in Diarrhea-Irritable Bowel Syndrome. BioMed Res. Int..

[20] Guo P., Furnary T., Vasiliou V., Yan Q., Nyhan K., Jones D.P., Johnson C.H., Liew Z. (2022). Non-targeted metabolomics and associations with per- and polyfluoroalkyl substances (PFAS) exposure in humans: A scoping review. Environ. Int..

[21] Xia D., Mo Q., Yang L., Wang W. (2022). Crosstalk between Mycotoxins and Intestinal Microbiota and the Alleviation Approach via Microorganisms. Toxins.

[22] Jia B.X., Lin H.K., Yang S., Li N., Yang D.Z., Wu A.B. (2023). Mycotoxin deoxynivalenol-induced intestinal flora disorders, dysfunction and organ damage in broilers and pigs. J. Hazard. Mater..

[23] He X., Zhou H.X., Fu X., Ni K.D., Li A.Z., Zhang L.T., Yin H.H., Jiang Q., Zhou X., Meng Y.W. (2024). Metabolomics study reveals increased deoxycholic acid contributes to deoxynivalenol-mediated intestinal barrier injury. Life Sci..

[24] Fan C.Y., Su D., Li X.J., Huang S., Zhang Y.D., Zheng N., Liu G.L., Liu Z., Cheng J.B. (2017). Effects of Aflatoxin B1 Individual, Binary or Tertiary Mixed with Ochratoxin A and Zearalenone on Intestinal Microorganism of Dairy Goat. China Anim. Husb. Vet. Med..

[25] Mariela S. (2020). *Streptococcus suis* Research: Progress and Challenges. Pathogens.

[26] Gao H., Kang X., Liu Z., Li Y., Bai L., Guo K., Huang M. (2026). Microbiota-bile acid-host axis dysregulation drives enteropathogenesis in *Escherichia coli* K99-induced neonatal calf diarrhea. Vet. Q..

[27] Nishikawa S., Ogawa Y., Eguchi M., Rambukkana A., Shimoji Y. (2018). Draft genome sequences of *Lawsonia intracellularis* swine strains causing proliferative enteropathy in Japan. Microbiol. Resour. Announc..

[28] Pusterla N., Gebhart C. (2013). *Lawsonia intracellularis* infection and proliferative enteropathy in foals. Vet. Microbiol..

[29] Ohta T., Kimura K., Katsuda K., Kobayashi H., Mikami O., Haritani M., Onodera T. (2016). Proliferative Enteropathy caused by *Lawsonia intracellularis* in Chickens. J. Comp. Pathol..

[30] Yong Y.J. (2023). Prevalence and associated risk factors for *Lawsonia intracellularis* infection in farmed rabbits: A serological and molecular cross-sectional study in South Korea. Front. Vet. Sci..

[31] Hwang J.M., Seo M.J., Yeh J.Y. (2017). *Lawsonia intracellularis* in the feces of wild rodents and stray cats captured around equine farms. BMC Vet. Res..

[32] Pusterla N., Mapes S., Gebhart C. (2012). Further investigation of exposure to *Lawsonia intracellularis* in wild and feral animals captured on horse properties with equine proliferative enteropathy. Vet. J..

[33] Tomanová I., Literák I., Klimeš J., Pavlačík L. (2003). *Lawsonia intracellularis* in Wild Mammals in the Slovak Carpathians. J. Wildl. Dis..

[34] Helm E.T., Burrough E.R., Gabler N.K., Leite F.L. (2026). *Lawsonia intracellularis* infection induces changes in microbial community function and composition associated with reduced pig growth and feed efficiency. Anim. Microbiome.

[35] White B.A., Lamed R., Bayer E.A., Flint H.J. (2014). Biomass Utilization by Gut Microbiomes. Annu. Rev. Microbiol..

[36] Liang J., Zhang R., Chang J., Chen L., Nabi M., Zhang H., Zhang G., Zhang P. (2024). Rumen microbes, enzymes, metabolisms, and application in lignocellulosic waste conversion—A comprehensive review. Biotechnol. Adv..

[37] Liang X., Li X., Mi N., Wu Y., Wu J., Chen H., Liu D. (2026). Early-Life Diarrhea Disrupts Antioxidant–Immune Homeostasis and Gut Microbiota in Suckling Calves. Biology.

[38] Yang Q.L., Huang X.Y., Zhao S.G., Sun W.Y., Yan Z.Q., Wang P.F., Li S.G., Huang W.Z., Zhang S.W., Liu L.X. (2017). Structure and Function of the Fecal Microbiota in Diarrheic Neonatal Piglets. Front. Microbiol..

[39] Li F., Yan M., Su D., Peng J., Wang X., Hao J., Ma T., Lin Y., Shi H. (2025). Integrated meta-omics reveals AFB1 dose-dependent remodeling of the rumen microbiome-virome-metabolome axis driving metabolic impairment in goats. Microbiome.

[40] Yanan G., Lu M., Huimin L., Jiaqi W., Nan Z. (2020). The Compromised Intestinal Barrier Induced by Mycotoxins. Toxins.

[41] Huang W., Zhang J., Miao C., Ying H., Zhang X., Song M., Cui Y., Wang X., Li Y., Cheng P. (2024). Aflatoxin B_1_-induced testosterone biosynthesis disorder via the ROS/AMPK signaling pathway in male mice. J. Agric. Food Chem..

[42] Adedara I.A., Nanjappa M.K., Farombi E.O., Akingbemi B.T. (2014). Aflatoxin B_1_ disrupts the androgen biosynthetic pathway in rat Leydig cells. Food Chem. Toxicol..

[43] Castagliuolo I., Karalis K., Valenick L., Pasha A., Nikulasson S., Wlk M., Pothoulakis C. (2001). Endogenous corticosteroids modulate Clostridium difficiletoxin A-induced enteritis in rats. Am. J. Physiol.-Gastrointest. Liver Physiol..

[44] Muir R.Q., Klocke B.J., Jennings M.S., Molina P.A., Hsu J.S., Kellum C.E., Alexander K.L., Lee G., Foote J.B., Lorenz R.G. (2023). Early life stress in mice leads to impaired colonic corticosterone production and prolonged inflammation following induction of colitis. Inflamm. Bowel Dis..

[45] Hussey M., Holleran G., Smith S., Sherlock M., McNamara D. (2017). The Role and Regulation of the 11 Beta-Hydroxysteroid Dehydrogenase Enzyme System in Patients with Inflammatory Bowel Disease. Dig. Dis. Sci..

[46] Coquenlorge S., Van Landeghem L., Jaulin J., Cenac N., Vergnolle N., Duchalais E., Neunlist M., Rolli-Derkinderen M. (2016). The arachidonic acid metabolite 11β-ProstaglandinF2α controls intestinal epithelial healing: Deficiency in patients with Crohn’s disease. Sci. Rep..

[47] Henkel F.D., Esser-von Bieren J. (2022). Not just “leuko” after all: Epithelial leukotriene production in type 2 immunity. Sci. Immunol..

[48] Wang J., Bakker W., Zheng W., de Haan L., Rietjens I.M.C.M., Bouwmeester H. (2022). Exposure to the mycotoxin deoxynivalenol reduces the transport of conjugated bile acids by intestinal Caco-2 cells. Arch. Toxicol..

[49] González-Arias C.A., Crespo-Sempere A., Marín S., Sanchis V., Ramos A.J. (2015). Modulation of the xenobiotic transformation system and inflammatory response by ochratoxin A exposure using a co-culture system of Caco-2 and HepG2 cells. Food Chem. Toxicol..

[50] Sun J., He Y., Chen R., Li Z., Li X., Han X., Li Y., Tang Z., Hu L. (2026). PTEN/PI3K/AKT Axis Mediates Aflatoxin B1-Induced Intestinal Injury via Dual Regulation of Apoptosis and Necroptosis in Jejunal Epithelial Cells. J. Agric. Food Chem..

[51] Xue L., Zhao W., Wang C., Ma Y., Tian J., Yang L., Ma L., Jiang Q., Chen Y., Tian X. (2026). Integrating multi-omics to characterize the dynamics of rumen microorganisms and metabolites in Angus cattle at different growth stages. Res. Vet. Sci..

[52] Zhuang Y., Gao D., Jiang W., Xu Y., Liu G., Hou G., Chen T., Li S., Zhang S., Liu S. (2025). Core microbe Bifidobacterium in the hindgut of calves improves the growth phenotype of young hosts by regulating microbial functions and host metabolism. Microbiome.

[53] Yang W.L., Shen Z.Q., Liang L.J., Zang Y.F., Dong Z.H., Bao L.D. (2025). Research Progress on Chemical Constituents and Pharmacological Effects of Wu Lingzhi. Chin. Tradit. Pat. Med..

[54] Soyoon B., Xuikui X., Sun M.B., Chanil P., Hee S.S. (2014). Trogopterins A-C: Three new neolignans from feces of *Trogopterus xanthipes*. Beilstein J. Org. Chem..

[55] Dubey V.S., Bhalla R., Luthra R. (2003). An overview of the non-mevalonate pathway for terpenoid biosynthesis in plants. J. Biosci..

[56] Weisburg W.G., Barns S.M., Pelletier D.A., Lane D.J. (1991). 16S ribosomal DNA amplification for phylogenetic study. J. Bacteriol..

[57] Chen H., Ma Y., Xu J., Wang W., Lu H., Quan C., Yang F., Lu Y., Wu H., Qiu M. (2024). Circulating microbiome DNA as biomarkers for early diagnosis and recurrence of lung cancer. Cell Rep. Med..

[58] Callahan B.J., McMurdie P.J., Rosen M.J., Han A.W., Johnson A.J., Holmes S.P. (2016). DADA2: High-resolution sample inference from Illumina amplicon data. Nat. Methods.

[59] Bolyen E., Rideout J.R., Dillon M.R., Bokulich N.A., Abnet C.C., Al-Ghalith G.A., Alexander H., Alm E.J., Arumugam M., Asnicar F. (2019). Reproducible, interactive, scalable and extensible microbiome data science using QIIME 2. Nat. Biotechnol..

[60] Schloss P.D., Westcott S.L., Ryabin T., Hall J.R., Hartmann M., Hollister E.B., Lesniewski R.A., Oakley B.B., Parks D.H., Robinson C.J. (2009). Introducing mothur: Open-source, platform-independent, community-supported software for describing and comparing microbial communities. Appl. Environ. Microbiol..

[61] Segata N., Izard J., Waldron L., Gevers D., Miropolsky L., Garrett W.S., Huttenhower C. (2011). Metagenomic biomarker discovery and explanation. Genome Biol..

[62] Han C., Shi C., Liu L., Han J., Yang Q., Wang Y., Li X., Fu W., Gao H., Huang H. (2024). Majorbio Cloud 2024: Update single-cell and multiomics workflows. iMeta.

[63] Yang H., Wu X., Li X., Zang W., Zhou Z., Zhou Y., Cui W., Kou Y., Wang L., Hu A. (2024). A commensal protozoan attenuates Clostridioides difficile pathogenesis in mice via arginine-ornithine metabolism and host intestinal immune response. Nat. Commun..

