# Supplementary material for: Integrated Gut Microbiota and Metabolome Analysis Reveals Dysbiosis and Metabolic Disturbances in Diarrhea of the Complex-Toothed Flying Squirrel (Trogopterus xanthipes): Possible Mycotoxin-Induction?
Source: Toxins (Basel). 2026 Aug 3;18(8):340. doi: 10.3390/toxins18080340 (PMC13517910; doi:10.3390/toxins18080340)
Supplement: Supplementary file 1 [file toxins-18-00340-s001.zip › toxins-4402111-supplementary figures.pdf]

Supplementary Materials: Integrated Gut Microbiota and Metabolome Analysis Reveals Dysbiosis and Metabolic Disturbances in Diarrhea of the Complex-Toothed Flying Squirrel (Trogopterus xanthipes): Possible Mycotoxin-Induction?

Lifeng Che, Bichen Miao, Lijuan Suo and Jie Tang *

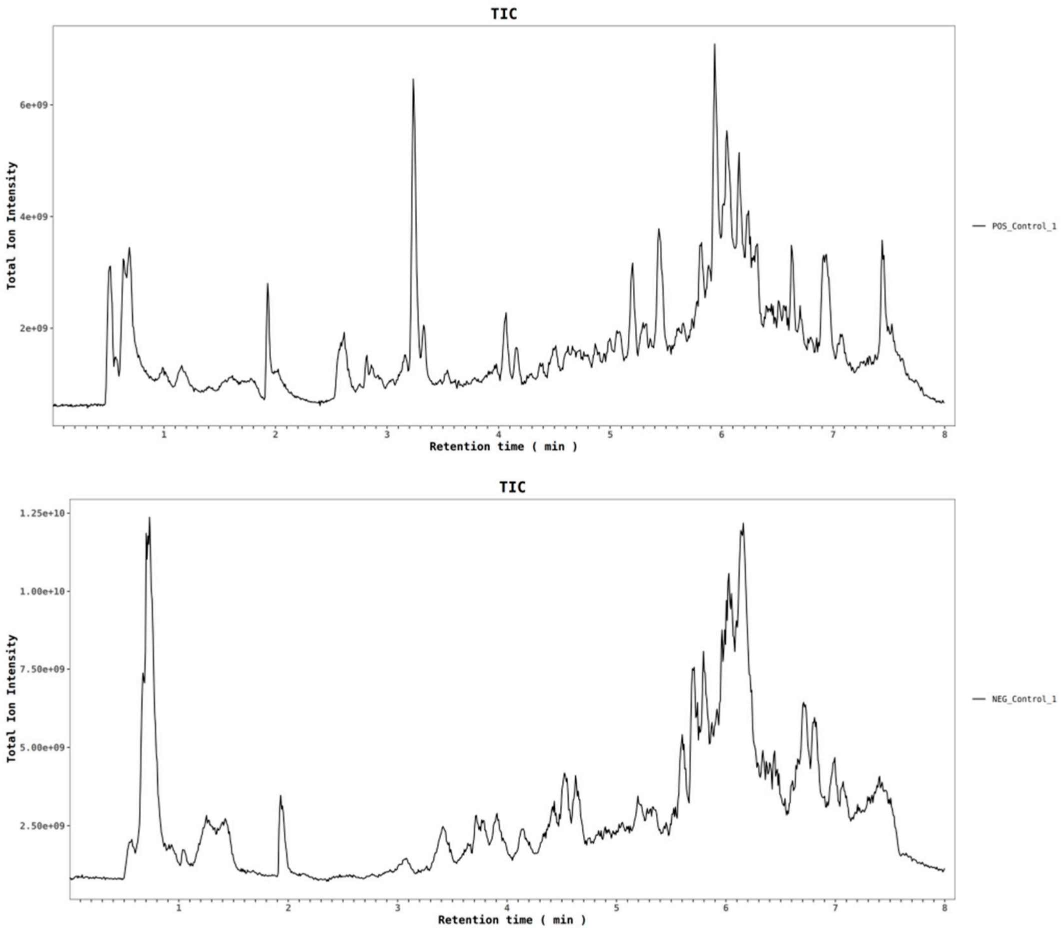

Figure S1. Total Ion Chromatography.

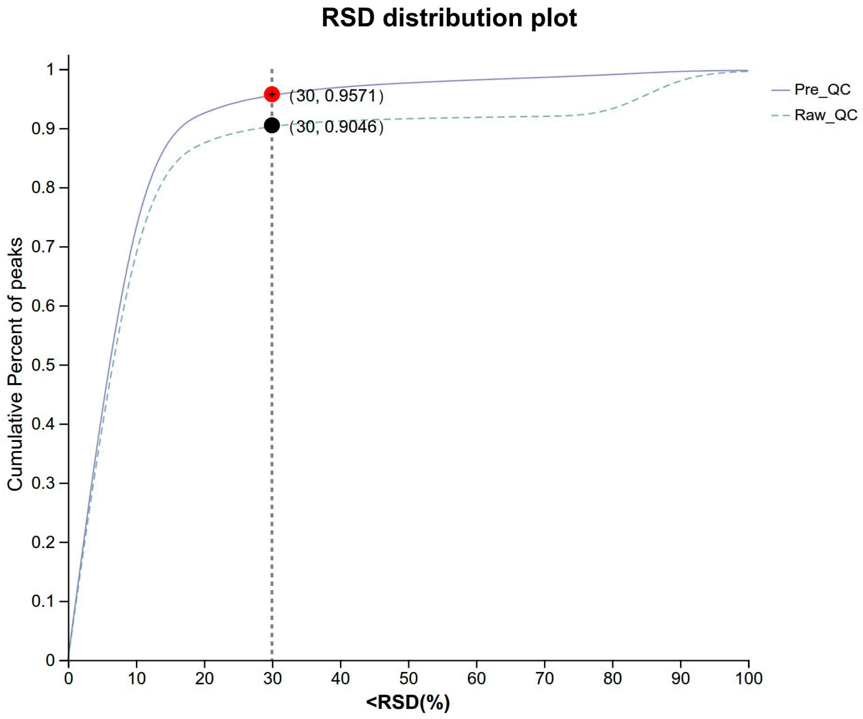

Figure S2. Cumulative RSD Distribution Plot of QC Samples.
